# Nitric oxide regulates photosynthesis and the capacity of the antioxidant system under water deficit and rehydration in *Lolium multiflorum*/*Festuca arundinacea* introgression forms

**DOI:** 10.3389/fpls.2025.1652482

**Published:** 2025-09-12

**Authors:** Dawid Perlikowski, Katarzyna Lechowicz, Sara Blicharz, Magdalena Arasimowicz-Jelonek, Adrianna Czapiewska, Izabela Pawłowicz, Arkadiusz Kosmala

**Affiliations:** ^1^ Plant Physiology Team, Institute of Plant Genetics, Polish Academy of Sciences, Poznan, Poland; ^2^ Integrative Plant Biology Team, Institute of Plant Genetics, Polish Academy of Sciences, Poznan, Poland; ^3^ Department of Plant Molecular Physiology, Institute of Bioorganic Chemistry, Polish Academy of Sciences, Poznan, Poland; ^4^ Department of Plant Ecophysiology, Institute of Experimental Biology, Faculty of Biology, Adam Mickiewicz University, Poznan, Poland

**Keywords:** drought tolerance, *Festuca arundinacea*, *Lolium multiflorum*, forage grasses, nitric oxide, photosynthesis, antioxidant system, recovery

## Abstract

Although numerous studies have already indicated the important roles of nitric oxide (NO) in adaptations of different plant species, including forage grasses, to water deficit conditions, the precise mechanisms of its action have not been fully recognized. Thus, the purpose of this work was to identify the key physiological traits and understand their relations with plant response to soil water deficit and subsequent rewatering under modulated NO content in *Lolium multiflorum*/*Festuca arundinacea* introgression forms distinct in the levels of drought tolerance. To reduce NO content in plant cells, NO scavenger, 2-phenyl-4,4,5,5-tetramethylimidazoline-1-oxyl-3-oxide (PTIO), was used. The obtained results clearly indicated a higher photosynthetic capacity of the plants with a decreased NO content on the 12th day of water deficit (12% of soil water content), which was manifested by a higher CO_2_ assimilation level. This phenomenon was associated with delayed stomata closure observed under these simulated conditions and resulted in a higher level of transpiration. Moreover, the plants with a lower NO content were characterized by a significantly higher water uptake in the early stages of water deficit progression, which could disturb their drought tolerance. Scavenging of NO also resulted in elevated H_2_O_2_ content, decreased activity of ascorbate peroxidase on the 14th day of water deficit (5% of soil water content) and subsequent rewatering, and a higher level of lipid peroxidation, which could impact cellular homeostasis of the analyzed introgression forms.

## 1 Introduction

Nitric oxide (NO) has recently become the subject of intense studies because of the growing evidence for its key role in the transmission of cellular signals in plants under adverse environmental conditions (Lau et al., 2021; Kumar and Puja Ohri, 2023; Khator et al., 2024). In general, NO is a gaseous, water- and lipid-soluble molecule that is produced under hormonal and environmental stimuli including drought (Kaya et al., 2020). In plants, NO biosynthesis might be associated with two different pathways (Astier et al., 2018). The best recognized is NO formation through nitrite (NO_2_^-^) reduction, which can be performed by enzymatic and non-enzymatic reactions. The enzymes involved in NO production from reduced nitrites are nitrate reductase (NR) (León and Costa-Broseta, 2020), nitrite NO reductase (NiNOR) (Cao et al., 2019), and peroxisomal xantine oxidoreductase (XOR) (Wang et al., 2010). The non-enzymatic pathway could generate NO under a high concentration of nitrite in an acidic environment and has been identified in *Hordeum vulgare* apoplast (Bethke et al., 2004). On the other hand, the second, less-studied route is associated with oxidation of aminated compounds such as L-arginine via nitric oxide synthase (NOS)-like enzymes, as well as polyamine or hydroxylamine (Corpas and Barroso, 2014; Peng et al., 2016; Rümer et al., 2009). Nitric oxide can react easily with reactive oxygen species (ROS), mainly superoxide anion radical (O_2_^•−^), to generate reactive nitrogen species (RNS) such as peroxynitrite (ONOO^−^), nitrogen dioxide (NO_2_), dinitrogen trioxide (N_2_O_3_), and many other molecules (Lindermayr and Durner, 2015). Thus, depending on the concentration, NO can play a dual role in plant cells. First of all, at optimal or slightly elevated levels, this molecule functions as an important signaling compound (Corpas et al., 2011; Khator et al., 2024; Kumari et al., 2024). However, for the second, its uncontrolled production can result in toxic levels creating nitrosative stress conditions (Casaretto et al., 2021; Kaya et al., 2024). Nitric oxide and RNS play a role in multiple physiological processes in plants, including shoot and root growth (Pissolato et al., 2020), stomatal closure (Yang et al., 2018), photosynthesis regulation (Ozfidan-Konakci et al., 2020), and formation of guard cells (de Sousa et al., 2020). Many studies have already indicated the role of NO in metabolic plant adaptation to drought stress (García-Mata and Lamattina, 2001; Desikan et al., 2002; Lamattina et al., 2003; Casaretto et al., 2021). However, the exact processes by which NO and its derivatives influence plant metabolism are still largely unknown. It has already been demonstrated that at least part of its functions is associated with post-translational modifications of proteins, leading to alterations or inhibition of protein functions and activities of enzymes (Lindermayr and Durner, 2015). The most recognized modification of proteins is S-nitrosylation, which is the direct modification of cysteine by NO itself (Wang et al., 2006). A second well-known protein modification is nitration of tyrosine residue directed by ONOO^−^ and NO_2_, which can be easily measured *in vitro*, being a good molecular indicator of nitrosative stress in cells (Corpas et al., 2008).

Nevertheless, the interaction between NO and ROS may also favor cell metabolism by regulating ROS contents and maintaining their homeostasis (Zhao et al., 2020; Samanta et al., 2024; Kandhol et al., 2024; Lee and Kim, 2024). The regulation might proceed directly by interacting with ROS molecules or indirectly by promoting transcriptional changes of genes, controlling several levels of plant metabolism and involved in plant defense, signal transduction, or antioxidant system (Del Castello et al., 2019).

The use of NO donors has been shown to decrease the levels of malondialdehyde (MDA)—an indicator of lipid peroxidation, O_2_^•−^, and hydrogen peroxide (H_2_O_2_) in plant cells during drought stress, thereby boosting the enzymatic or non-enzymatic antioxidant system (Majeed et al., 2020; Gan et al., 2015; Li et al., 2019). Hydrogen peroxide, as a signaling molecule, plays a crucial role in regulating plant metabolism. Additionally, NO can promote the production of H_2_O_2_ (Shi et al., 2015). These molecules are linked to the regulation of osmolyte content, abscisic acid (ABA)-induced antioxidant activity, stomatal movement, and enhancement of photosynthesis (Wang et al., 2021; Bright et al., 2006; Liao et al., 2012a, 2012b).

Another physiological role of NO is associated with the regulation of phytohormone activities. Nitric oxide has been recognized to crosstalk with many plant hormones such as cytokinins, auxins, ABA, ethylene, or jasmonic acid, mitigating their functions in many physiological processes associated also with abiotic stress response (Durbak et al., 2012; Freschi, 2013; Simontacchi et al., 2013). On the other hand, though ABA plays an important function in the signal transduction in plant response to abiotic stress factors, contradictory results of numerous experiments conducted to clarify the mechanisms of ABA/NO cooperation have not enabled a full understanding of these processes yet (García-Mata and Lamattina, 2001; Desikan et al., 2002; Neill et al., 2002; Lamattina et al., 2003; Lozano-Juste and León, 2010; León et al., 2014; Casaretto et al., 2021).

Most recent studies have focused on the application of exogenous NO to plants in the form of synthetic NO donors. However, the amount of physiologically active NO released from the donor compound depends on its chemical structure, concentration, application method, and plant material, which consequently causes different results to be observed (Floryszak-Wieczorek et al., 2006; Tan et al., 2013; Salahuddin et al., 2017; Antoniou et al., 2020; van Meeteren et al., 2020). This indicates the need for additional research to optimize experimental strategies in this field (Leite et al., 2019). Another approach, adopted in the present study, is based on the use of endogenous NO scavengers such as 2-4-carboxyphenyl-4,4,5,5-tetramethylimidazoline-1-oxyl-3-oxide (PTIO) or cPTIO (García-Mata and Lamattina, 2002; Wu et al., 2017). The obtained results clearly indicated an important role of NO generation in plants under drought conditions. Its higher content was shown to be mainly associated with the promotion of stomatal closure in the presence of ABA (García-Mata and Lamattina, 2001; Desikan et al., 2002; Lamattina et al., 2003; Neill et al., 2002). Similar findings were also obtained in the research on the mechanisms of NO biosynthesis, especially in the case of NR and NOS-like activities (Planchet et al., 2005; Corpas et al., 2009; Astier et al., 2018; Khator et al., 2024). However, other authors postulated a contradictory theory in which NO is not required to regulate stomatal aperture in response to ABA (Lozano-Juste and León, 2010; León et al., 2014), and some others even suggested that NO can be a negative regulator of ABA (Wang et al., 2015). Aside from ABA, NO can also interact with other important plant signaling molecules, including phytohormones such as cytokinins (Shao et al., 2010; Negi et al., 2010), auxins (Remy et al., 2013), and jasmonic acid (Liu et al., 2005; Palmieri et al., 2008; Shan et al., 2015). It has been proposed that NO also has the ability to directly and indirectly interact with other signal transduction components, such as protein kinases or secondary messengers such as Ca^2+^ (Khan et al., 2023; Mir et al., 2022; Courtois et al., 2008).

Although the functions of NO in the response of plants to environmental stimuli have been elucidated even with respect to forage grasses (Hatamzadeh et al., 2015; Li et al., 2016; Perlikowski et al., 2022), the precise mechanisms of its action have not been fully recognized. Thus, the purpose of this work was to identify the physiological traits of forage grasses that are modulated when NO content is reduced under drought and subsequent rehydration conditions. We hypothesize that a depletion of endogenous NO may significantly alter crucial physiological processes associated with a potential of drought tolerance in *Lolium–Festuca* hybrids, including photosynthesis as well as antioxidative capacity. To verify this hypothesis, we analyzed two well-characterized *Lolium multiflorum*/*Festuca arundinacea* (Lm/Fa) introgression forms, distinct in the levels of drought tolerance (Perlikowski et al., 2014) with respect to their response to soil water deficit and subsequent rewatering, a low-drought-tolerant genotype (LDT), and a high-drought-tolerant genotype (HDT). The experiments were conducted both in conditions where endogenous NO production was disturbed in plants by the application of PTIO and in control conditions in which plants were not modified with respect to NO content. To describe the photosynthetic efficiency of plants, i) gas exchange parameters (stomatal conductance, transpiration, and CO_2_ assimilation); ii) maximum quantum efficiency of photosystem II (F_v_/F_m_), an important parameter of chlorophyll fluorescence indicating the efficiency of photosynthetic light reactions; and iii) the activity of chloroplastic fructose-1,6-bisphosphate aldolase (pFBA), a crucial enzyme of the Calvin cycle, were analyzed. To describe the antioxidant efficiency of plants, i) the contents of ROS (O_2_^•−^ and H_2_O_2_), ii) the activity of ascorbate peroxidase (APX) and catalase (CAT), and iii) the content of MDA to recognize levels of lipid peroxidation were analyzed. Relative water content (RWC) to reveal leaf hydration status, electrolyte leakage (EL) to evaluate cellular membrane integrity, and the content of ABA (a phytohormone involved in numerous metabolic pathways associated with drought response) were also measured.

## 2 Materials and methods

### 2.1 Plant materials

The plant materials consisted of two tetraploid (2*n* = 4× = 28) *L. multiflorum*/*F. arundinacea* (Lm/Fa) introgression forms that are distinct in their levels of drought tolerance: the HDT form and the LDT form (Perlikowski et al., 2014). The analyzed introgression forms were generated earlier through four backcrossing series of F_1_ pentaploid (5×) *L. multiflorum* (4×) × *F. arundinacea* (6×) hybrid to tetraploid *L. multiflorum* (4×). These forms were germinated from single seeds, which were from the collection of the Institute of Plant Genetics, Polish Academy of Sciences. The plants were selected as described by Perlikowski et al. (2014). The analysis of their genomic structure using genomic *in situ* hybridization confirmed the identity of both introgression forms (data unpublished). The introgression forms as well as their parental species are not under the species conservation law; thus, formal national legislation is not required. All the experiments performed with these forms were in accordance with relevant institutional guidelines and regulations. These two Lm/Fa forms have been used previously as models in several experiments associated with plant response to soil water deficit (Perlikowski et al., 2014, 2016a, 2016b, 2019, 2022, 2023).

### 2.2 Experimental setup

Prior to the experiment, individual tillers from pre-cultivated plant clumps were isolated for each introgression form. Three sets (biological replications) of 10 healthy tillers in a similar growth stage were placed in freshly prepared 2.5 dm^3^ pots filled with a 1:3 mixture of sand and peat. Each form was represented by 20 pots, and four parallel different experimental groups were set up: i) plants watered without PTIO application, ii) plants watered with PTIO application, iii) plants subjected to water deficit without PTIO application, and iv) plants subjected to water deficit with PTIO application. Plants were then acclimated for 2 months in a growth chamber with stable environmental conditions: temperature 22°C, 16 h photoperiod, 200 µmol m^−2^ s^−1^ photosynthetic photon flux density (PPFD), air humidity 55 – 60%, and daily watering with a modified Long Ashton nutrition solution (Garczyński and Sokołowska-Garczyńska, 2007; Perlikowski et al., 2019, 2022, 2023). During this period, dozens of new young tillers were developed by vegetative reproduction, and these fresh tillers were used during the experiment. Following an additional week of plant acclimation, PTIO applications were initiated. Plants subjected to PTIO treatment were watered daily with 20 mL of 200 µM PTIO solution, and an additional 2 mL of PTIO solution was sprayed onto the leaves daily. Conversely, plants without PTIO treatment received equal amounts of watering and spraying with distilled water. It was previously determined that PTIO in the water solution remains stable for at least 20 h at room temperature (Li, 2017). After a 5-day treatment period, half of the plants in each group underwent water deficit conditions, while the remaining half were continuously watered to maintain 50% of soil water content (SWC) until the end of the experiment. The air humidity was reduced to 40%–45% under water deficit progression. Even under water deficit conditions, plants receiving PTIO treatment continued to be provided with the appropriate application, while the untreated plants received an equivalent amount of water.

Throughout the experiment, SWC levels were monitored by weighing and adjusting the pots daily to equal water level to ensure consistent SWC levels in each pot within the drought-treated groups. SWC was calculated according to the formula: ((WM − DM)/WM × 100), where WM indicated watered medium and DM indicated dry medium. According to the differences in the amount of lost water, the water uptake (WU) parameter was calculated. After 14 days of water deficit progression, when SWC reached 5%, the plants were rehydrated to reach maximum SWC and were maintained under well-watered conditions for 7 days. Analyses of RWC, EL, leaf gas exchange, and chlorophyll fluorescence were conducted every second day to establish the time point at which plants started to react to water deficit. The 12th day of water deficit (12D WD; 12% of SWC) was shown to be the initial day, at which at least a part of the plant experimental groups started to react to water deficit conditions. Between the 2nd and 10th day of water deficit progression, no statistical differences in RWC, EL, gas exchange, and chlorophyll fluorescence parameters were observed in the plants, as compared to the control conditions (**Supplementary Table S1**). Analyses of NO content, pFBA activity, ABA assay, MDA content, ROS content, and activity of antioxidant enzymes were conducted at four specific time points during the experiment: before initiation of water deficit conditions (C; watered conditions), after 14 days of water deficit (14D WD; 5% of SWC; the time point at which all the experimental plant groups experienced water deficit with respect to RWC, EL, and gas exchange), after 1 day (1D RH; initial rehydration stage) rewatering, and after 7 days (7D RH; advanced rehydration stage) of rewatering.

### 2.3 Leaf-specific nitric oxide visualization

As previously reported (Perlikowski et al., 2022), nitric oxide was detected by measuring the fluorescence of the products produced following the reaction with the fluorescent dye 4,5-diaminofluorescein diacetate (DAF-FM DA) (Sigma - St. Louis, MO, USA, currently member of Merck Group, Darmstadt, Germany). To determine the NO content in the leaves, 10 discs measuring 0.8 cm in diameter were excised from the middle section of the second, fully developed leaves (five leaves per one biological replication). The experimental group consists of three biological replications collected from three separate plants. These discs were then treated with 1 mL of incubation buffer containing 20 µM of DAF-FM DA in 10 mmol of HEPES-KOH at pH 7.4. Subsequently, the samples were incubated for 1 h in darkness, followed by two washes with 10 mmol of HEPES-KOH at pH 7.4. After the final wash, the samples were finely homogenized in 1 mL of HEPES buffer and centrifuged at 900*g* at room temperature. Fluorescence measurements were conducted using a Fluorescence Spectrophotometer F-2500 (Hitachi, Japan) with excitation at 470 nm and emission at 515 nm. The recorded fluorescence values were normalized against the autofluorescence of leaf discs that were cut from the analyzed plants but were only incubated in HEPES-KOH buffer without the DAF-FM DA dye.

Cross-sections of leaf samples were obtained by the midsection of the second, fully expanded leaves in three replicates (Perlikowski et al., 2022). Twenty cross-sections, each 0.1 mm thick, were immersed in 1 mL of 20 µM DAF-FM DA solution in 10 mmol of HEPES-KOH at pH 7.4 and left to incubate for 1 h in darkness. Following the incubation period, the buffer was aspirated, and the cross-sections were rinsed twice with 10 mmol of HEPES-KOH at pH 7.4. Leaf sections were mounted on glass slides and examined using the AXIO Image M2 microscope (Carl Zeiss in Gottingen, Germany). This microscope was equipped with a motorized stage, the Colibri LED-based fluorescent light source, and the AxioCam ICc5 camera. The fluorescence of DAF-FM DA was excited using a 470-nm blue LED, and imaging was conducted using the Zeiss filter set No. 38 HE (excitation BP 470/40, beam splitter FT 495, emission BP 525/50). The data obtained were recorded and analyzed using the ZEN version 2.3 (blue edition) software (www.zeiss.com) internally developed by Carl Zeiss in Jena, Germany.

### 2.4 Relative water content and electrolyte leakage

Analyses were performed following established protocols as described in our previous publications (Kosmala et al., 2012; Perlikowski et al., 2014), and the experiments were conducted on the middle section cut from the second, fully expanded leaves in 10 replicates per time point.

The RWC was computed using the following formula: RWC% = (FW − DW)/(TW − DW) × 100, where FW represented leaf fresh weight, DW indicated leaf dry weight, and TW represented leaf turgid weight. The EL parameter was determined by the formula: L1/L2 × 100, where L1 denoted electrolyte leakage of freshly collected leaves and L2 represented the full electrical conductivity of these leaves disrupted by liquid nitrogen. The EL measurements were carried out using the EC215 Conductivity Meter (Hanna Instruments, Leighton Buzzard, UK).

### 2.5 Abscisic acid content

The analysis of ABA content was conducted utilizing the “Plant hormone abscisic acid (ABA) ELISA Kit” (CUSABIO; www.cusabio.com, CSB-E09159Pl). Each time point in the experiment was replicated three times biologically, following the specified protocol for accurate measurements (Perlikowski et al., 2022).

### 2.6 Chlorophyll fluorescence

Chlorophyll *a* fluorescence parameters, including (F_v_/F_m_)—the maximum quantum efficiency of photosystem II photochemistry, were calculated based on measurements taken with the Handy PEA fluorimeter (Hansatech Instruments Ltd., King’s Lynn, England) at midday (Kosmala et al., 2012; Perlikowski et al., 2014) on the second, fully developed leaves in 10 replicates per time point. Leaves were adapted to the dark for 30 min with special clips provided by the manufacturer.

### 2.7 Gas exchange

Gas exchange parameters—CO_2_ assimilation rate (P_N_), stomatal conductance (*g_s_*), transpiration (E), and internal CO_2_ concentration (C_i_)—were computed from measurements obtained with the second, fully developed leaves in five replicates during midday using the LI-6400XT Portable Photosynthesis System from LI-COR (USA) with a RGB-0241 chamber (irradiance = 1,000 µmol m^−2^ s^−1^, RGB lamps, CO_2_ reference concentration 380 µmol mol^−1^, air flow rate through the assimilation chamber 400 µmol s^−1^, relative humidity 30%, and chamber temperature 20 °C) (Kosmala et al., 2012; Perlikowski et al., 2014).

### 2.8 Activity of chloroplast fructose-1,6-bisphosphate aldolase

The activity of pFBA was assessed using a modified Sibley–Lehninger method (Sibley and Lehninger, 1949; Willard and Gibbs, 1968). Native chloroplast proteins were extracted following a previously established protocol, with slight modifications (Perlikowski et al., 2016a; Lechowicz et al., 2020). One gram of frozen leaf tissue collected from three biological replicates of tissue harvested only from the second, fully developed leaves was homogenized and suspended in 4 mL of chloroplast isolation buffer (CIB) (Sigma - St. Louis, MO, USA, currently member of Merck Group, Darmstadt, Germany) containing 0.1% BSA. The samples were filtered through a Sefar Nitex mesh (100 μm) and centrifuged at 200*g* for 3 min at room temperature. The supernatant was transferred to new tubes and centrifuged again at 900*g* for 15 min at room temperature. The resulting pellet was washed twice with 1 mL of CIB without BSA. The chloroplast pellet was then dissolved in 500 μL of protein isolation buffer (0.1 mol Na_2_HPO_4_, 3% Triton X - 100), vortexed, and centrifuged at 21,500*g* for 10 min at room temperature. The supernatant was used to measure pFBA activity. For the assay, 100 μL of the sample was mixed with the isolation buffer. In three tubes per sample, 100 μL of 0.06 mol fructose-1,6-bisphosphate was combined with 140 μL of incubation buffer (0.05 mol of 2,4,6-trimethylpyridine, 0.08 mol of hydrazine sulfate, 0.3 mmol of sodium iodoacetate, pH 7.4) and pre-incubated at 30°C for 10 min in a water bath. A reference tube containing an additional 300 μL of 10% trichloroacetic acid (TCA) was prepared. After pre-incubation, 50 μL of the chloroplast extract was added to each tube, mixed, and incubated at 30°C for 2 h. The reaction was terminated by adding 300 μL of 10% TCA, and the samples were chilled on ice and centrifuged. From each tube, 100 μL of the supernatant was mixed with 100 μL of 0.75 mol NaOH and incubated at room temperature for 10 min. Subsequently, 100 μL of 0.1% 2,4-dinitrophenylhydrazine was added, followed by incubation at 30°C for 10 min. Finally, 700 μL of 0.75 mol NaOH was added and mixed, and absorbance at 540 nm was measured within the next 10 min. A standard curve was generated using serial dilutions of 0.1 mmol of D-glyceraldehyde (Perlikowski et al., 2016a). The pFBA activity was calculated based on the amount of trioses produced by pFBA in 1 g of fresh sample per hour. Each time point in the experiment was replicated three times biologically.

### 2.9 Activity of the antioxidant enzymes

Ascorbate peroxidase activity was assessed using the Ascorbate Peroxidase Microplate Assay Kit (Cohesion Biosciences, CAK1052), following the manufacturer’s guidelines. Finely ground leaf tissue from three biological replicates of tissue harvested only from the second, fully developed leaves per time point was utilized. One unit of APX activity was defined as the quantity of enzyme required to oxidize 1 µmol of ascorbic acid per minute.

Catalase activity was measured following the protocol described by Dhindsa et al. (1981) and Lechowicz et al. (2020), with some modifications. Leaf extracts were prepared from 0.2 g of homogenized leaf tissue harvested only from the second, fully developed leaves, using three biological replicates per time point. The tissue was homogenized in 1 mL of 50 mmol KH_2_PO_4_ buffer at pH 7.0, then centrifuged at 14,000*g* for 20 min at 4°C to obtain the supernatant. CAT activity was assessed spectrophotometrically by monitoring the absorbance change at 240 nm, which corresponds to the decomposition of H_2_O_2_. One unit of CAT activity was defined as the amount of enzyme required to decompose 1 μmol of H_2_O_2_ per minute, calculated using an extinction coefficient of 45.2 mmol^−1^ cm^−1^.

The protein concentration in the samples was quantified using Bradford’s method (Bradford, 1976). Each enzymatic activity value was then normalized to the soluble protein content in the sample and expressed per milligram of protein. Spectrophotometric readings were conducted with the Synergy HTX Multi-Mode Reader (BioTek Instruments, Winooski, Vermont, USA).

### 2.10 Superoxide anion radical and hydrogen peroxide content

The superoxide radical content was measured spectrophotometrically following the methods described previously (Doke, 1983; Arasimowicz et al., 2009; Lechowicz et al., 2020). Leaf discs, each approximately 0.8 cm in diameter, were excised from the middle section of the second, fully expanded leaves and incubated for 1 h in the dark with 3 mL of a pH 7.8 mixture containing 0.05 mol of KH_2_PO_4_/K_2_HPO_4_, 0.1 mmol of EDTA, 10 mmol of NaN_3_, and 0.05% nitroblue tetrazolium (NBT). NBT was reduced by O_2_^•−^ to form blue diformazan. After incubation, the samples were heated at 85°C for 15 min. Once cooled, the absorbance was read at 580 nm. The O_2_^•−^ content was expressed as the absorbance per gram of fresh leaf weight. Each time point in the experiment was replicated four times biologically.

The hydrogen peroxide content was assessed using the titanium (Ti^4+^) method (Becana et al., 1986; Arasimowicz-Jelonek et al., 2013; Lechowicz et al., 2020). Plant leaf tissue harvested only from the second, fully developed leaves, weighing 0.4 g, was homogenized in three biological replicates using a TissueLyser (Qiagen, Hilden, Germany) with 1.5 mL of chilled 0.1 M potassium phosphate buffer (pH 7.8). The homogenate was then centrifuged at 14,000*g* for 25 min at 4°C to obtain the supernatant. The reaction mixture, comprising 400 µL of tissue extract, 600 µL of potassium phosphate buffer, and 500 µL of titanium reagent (0.6 mmol of PRL and 0.6 mmol of PTO in a 1:1 ratio), was prepared in two technical replicates per sample and incubated at room temperature for 10 min. Following incubation, absorbance was measured at 508 nm using an Ultrospec 1100 pro spectrophotometer (Amersham Biosciences, Chalfont St. Giles, UK). The H_2_O_2_ concentration was determined using a standard curve and expressed as µmol H_2_O_2_ per gram of fresh weight. Each time point in the experiment was replicated three times biologically.

### 2.11 Malondialdehyde content

The MDA content was determined based on the level of thiobarbituric acid-reactive substances (TBARS) in three separate samples. TBARS levels were quantified spectrophotometrically at wavelengths of 532 nm and 600 nm, following a slightly modified version of the method of Heath and Packer (1968) as described by Lechowicz et al. (2020). The TBARS amount was calculated using the formula: TBARS (µmol) = (*A*_532_ − *A*_600_)/155, where 155 represents the extinction coefficient, and the results were expressed per gram of fresh weight. Each time point in the experiment was replicated three times biologically from tissue harvested only from the second, fully developed leaves.

### 2.12 Statistical analysis

The statistical evaluations were executed using STATISTICA version 13.0 (www.statsoft.pl) software (StatSoft, Tulsa, OK, USA). A two-way analysis of variance (ANOVA) was carried out, considering genotype and time point as classification factors. Fisher’s least significant difference (LSD) test at a *p*-value of 0.05 was used to evaluate differences between the subjects over the duration of the experiment. Homogeneous groups identified by the test are indicated by the same letters on the graphs.

## 3 Results

### 3.1 Soil water content and water uptake

**Figure 1:**
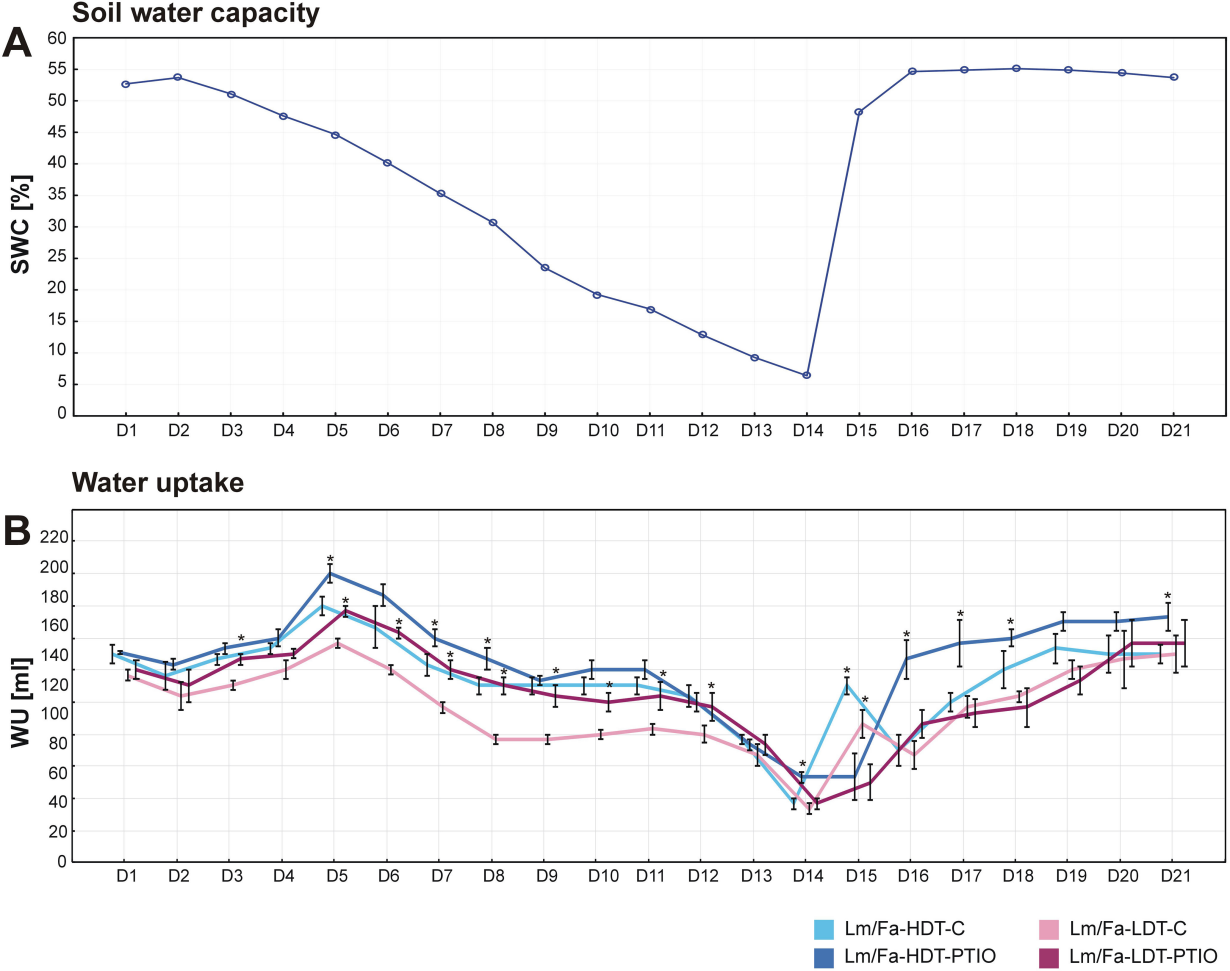
Evaluation of soil water content (SWC) **(A)** and water uptake (WU) **(B)** during the experiment in the analyzed introgression forms. Error bars indicate standard errors. Asterisks indicate the statistical significance between 2-phenyl-4,4,5,5-tetramethylimidazoline-1-oxyl-3-oxide (PTIO)-treated and control non-treated (C) introgression forms according to Student’s *t*-test (*p* = 0.05).

During the 14 days of the water deficit period, SWC was reduced from 50% to 5%, while after rewatering (recovery period), its level was restored to the initial values (**Figure 1A**). It was observed that starting from the 3rd day of the experiment (in the case of the LDT form) and from the 5th day (in the case of the HDT form), the introgression forms, not treated with PTIO (control forms) and those treated with NO scavenger, began to differentiate with respect to WU, indicating that the PTIO-treated plants were characterized by higher WU compared to the control plants not treated with PTIO. Moreover, these differences were visible until the 8th day and the 12th day of the water deficit period, for the HDT and the LDT forms, respectively. Significantly similar differences between the control plants and the PTIO-treated plants were also observed during the recovery period, but only in the case of the HDT introgression form (**Figure 1B**). However, during the first day of recovery, we could observe that the plants not treated with PTIO increased their WU, while in PTIO-treated plants, it was noticeable after 2 days of rewatering (**Figure 1B**).

### 3.2 Nitric oxide content

**Figure 2:**
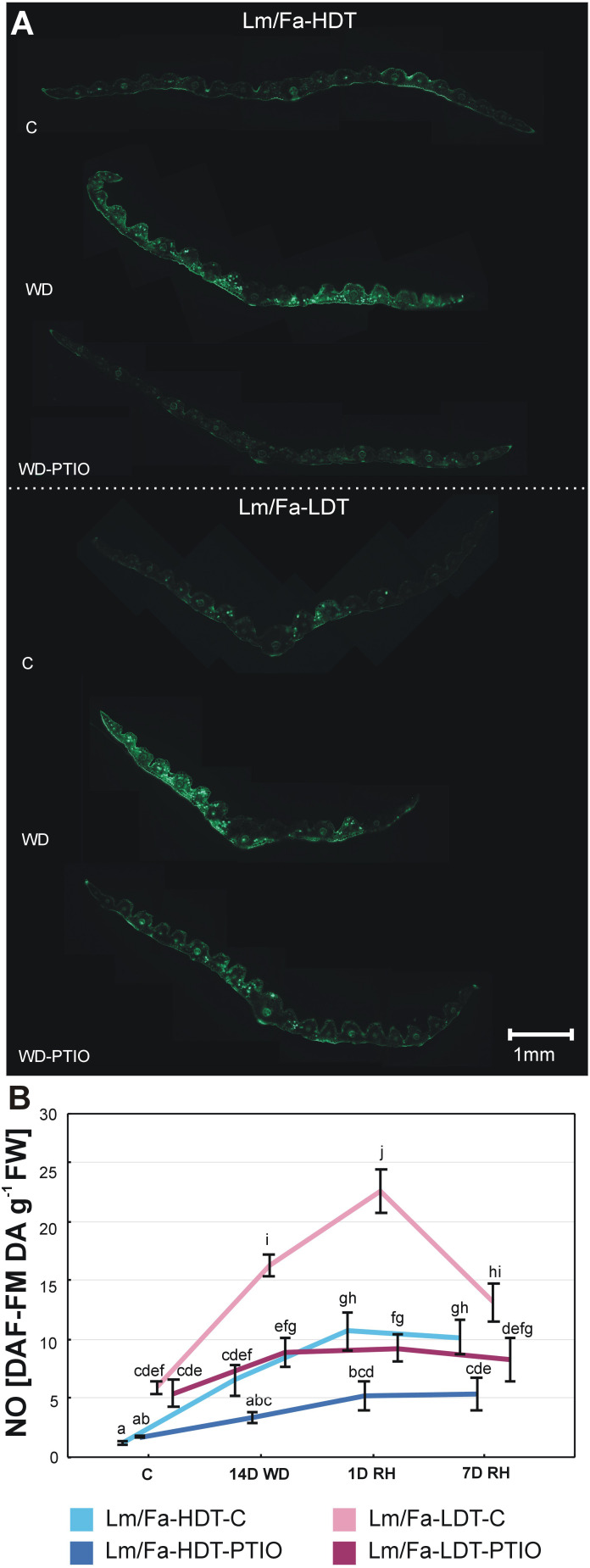
Bio-images represent the presence of endogenous NO accumulation in leaf cross-sections in the control conditions without PTIO (C), on the 14th day of water deficit without PTIO (WD), and on the 14th day of water deficit with PTIO (WD-PTIO). Green signal indicates NO accumulation **(A)**. The level of nitric oxide (NO) content in the leaves of the analyzed introgression forms in control conditions (C), on the 14th day of water deficit (14D WD), on the 1st day of rewatering (1D RH), and on the 7th day of rewatering (7D RH) **(B)**. Error bars represent the standard errors. Homogeneous groups are indicated by the same letter according to the Fisher-LSD test (*p* = 0.05). HDT, high-drought-tolerant form; LDT, low-drought-tolerant introgression form; C, control introgression forms not treated with PTIO. PTIO, introgression forms treated with PTIO.

Water deficit significantly increased NO content in both the analyzed Lm/Fa introgression forms (**Figure 2**). NO was located preferentially in the mesophyll and bundle sheath cells (**Figure 2A**). The LDT form was characterized by significantly higher NO content than the HDT form, both in the control (~392%) and stress conditions (~150%) (**Figure 2B**, **Supplementary Table S2**). However, the highest values for NO content were observed after rewatering, compared to the control (~283%). In the HDT form, it remained elevated up to the 7th day of rewatering, while in the LDT form, it decreased significantly but was still higher than in the control conditions. The application of PTIO significantly reduced the accumulation of NO in both forms on the 14th day of the water deficit and recovery periods. In the LDT form treated with PTIO, a significantly higher NO content was identified only after 1 day of rewatering (~70%), compared to control conditions, while in the HDT form treated with PTIO, a significantly higher NO content was identified after 7 days of rewatering (~214%) (**Figure 2B**, **Supplementary Table S2**).

### 3.3 Leaf water status and membrane stability

**Figure 3:**
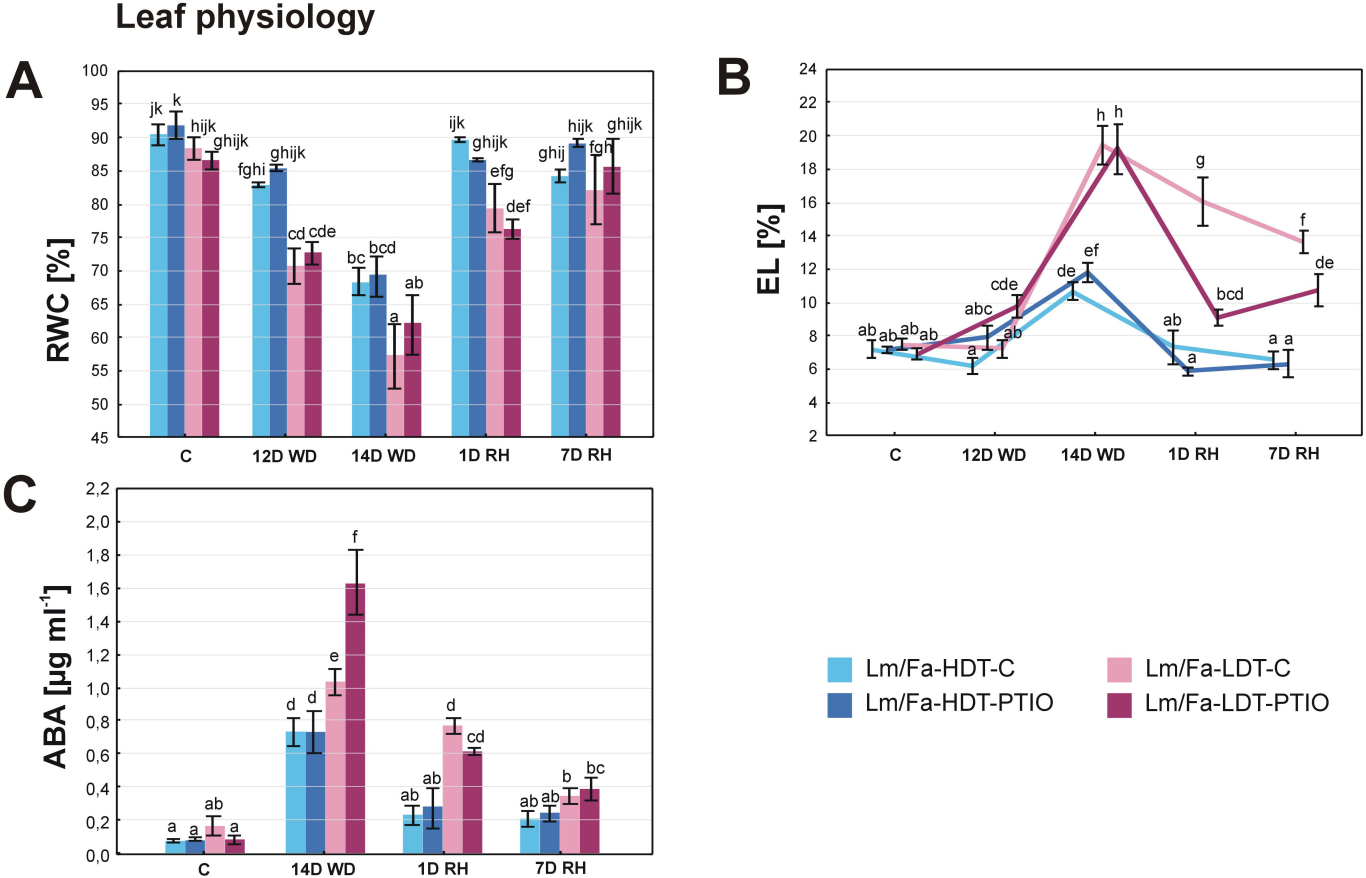
Relative water content (RWC) **(A)**, electrolyte leakage (EL) **(B)**, and content of abscisic acid (ABA) **(C)** in the analyzed introgression forms in control conditions **(C)**, on the 12th day of water deficit (12D WD), on the 14th day of water deficit (14D WD), on the 1st day of rewatering (1D RH), and on the 7th day of rewatering (7D RH). Error bars represent the standard errors. Homogeneous groups are indicated by the same letter according to the Fisher-LSD test (*p* = 0.05). HDT, high-drought-tolerant form; LDT, low-drought-tolerant introgression form; C, control introgression forms not treated with PTIO. PTIO, introgression forms treated with PTIO.

The first statistically significant differences in RWC and EL between the plants in the control conditions and the plants cultivated under water deficit conditions started to be visible on the 12th day of the experiment. Thus, differences in RWC between the HDT and LDT introgression forms were revealed on the 12th (~17%) and 14th day (~20%) of water deficit and 1 day after rewatering (~13%), indicating higher RWC in the HDT form not treated with PTIO. However, PTIO treatment did not have any impact on RWC level (**Figure 3A**, **Supplementary Table S2**).

Under drought conditions, an increased level of the EL parameter (**Figure 3B**) was observed. However, on the 12th day of water deficit, an elevated value (~42%) of this parameter was observed only in the LDT form treated with PTIO. After 14 days of water deficit, differences between the HDT and LDT introgression forms were also observed, but without any impact of the PTIO treatment. Rewatering triggered a membrane regeneration process in all the plants, as decreased EL was observed already after 1 day of rewatering. The HDT form, both the PTIO-treated and non-treated, reached full recovery of EL to the values observed in control conditions after 1 and 7 days of rewatering, while the form LDT reduced EL after 1 day of rewatering, but even after 7 days, it did not reach the control values. Meanwhile, the LDT form treated with PTIO also reduced EL during recovery, and it was significantly more efficient than in the case of plants not treated with PTIO (**Figure 3B**, **Supplementary Table S2**).

### 3.4 Abscisic acid content

Under drought, ABA content increased in all the analyzed plants. However, the impact of PTIO treatment on ABA accumulation was indicated only in the LDT form on the 14th day of water deficit conditions, displaying 58.5% higher ABA content in LDT-PTIO plants. When rewatering was started, ABA content returned to the values observed in control conditions in the HDT form, while in the LDT form, it decreased significantly but remained slightly elevated. After 7 days of rewatering, ABA content reached control values in the LDT form not treated with PTIO, while in the LDT form treated with this NO scavenger, the content of this hormone was higher (~399%), compared to control conditions (**Figure 3C**, **Supplementary Table S2**).

### 3.5 Leaf gas exchange

**Figure 4:**
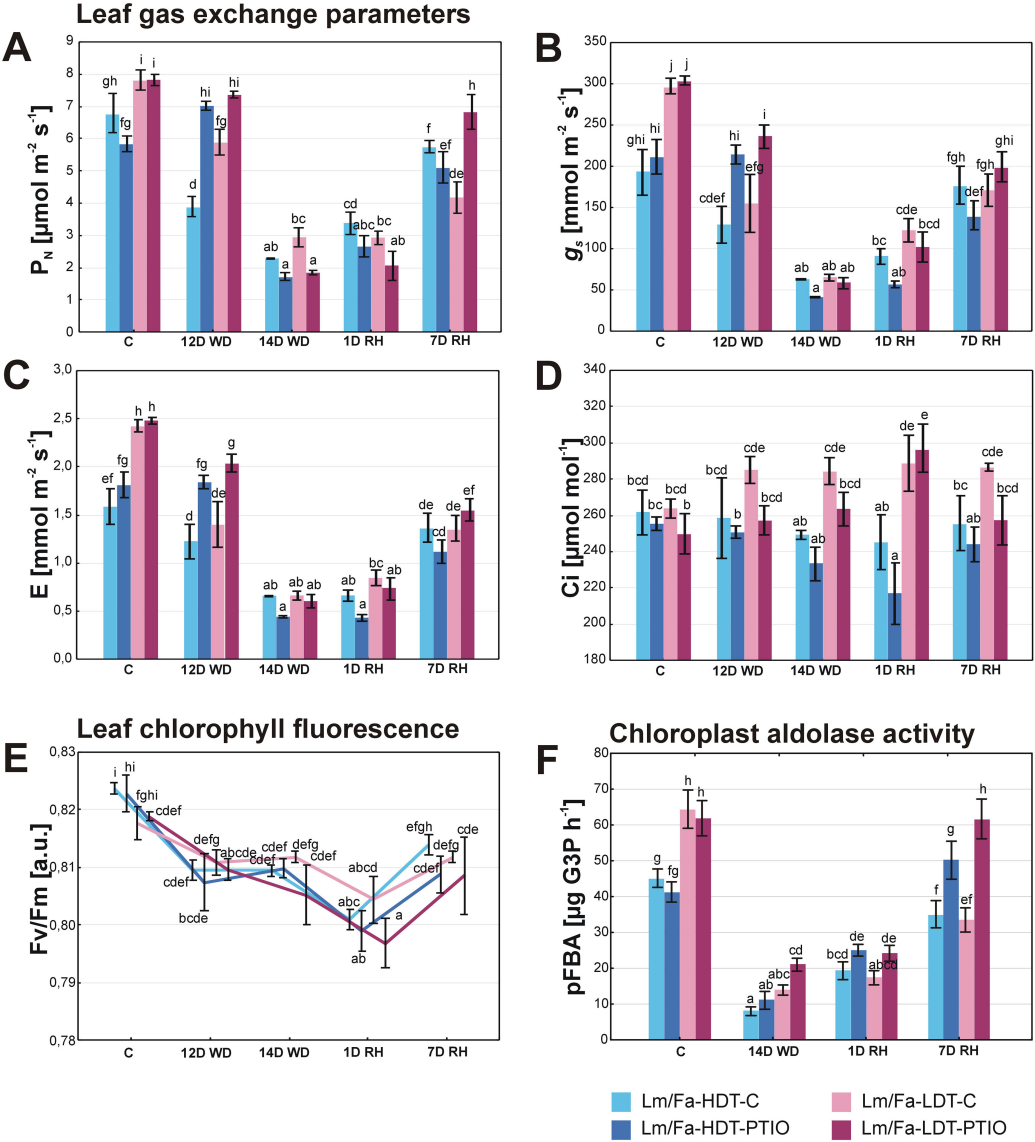
Leaf photosynthesis parameters: CO_2_ assimilation rate (P_N_) **(A)**, stomatal conductance (*g_s_*) **(B)**, transpiration (E) **(C)**, internal CO_2_ concentration (C_i_) **(D)**, maximum quantum efficiency of photosystem II photochemistry (F_v_/F_m_) [arbitrary units] **(E)**, and chloroplast aldolase activity (pFBA) **(F)** in the analyzed introgression forms in control conditions (C), on the 12th day of water deficit (12D WD), on the 14th day of water deficit (14D WD), on the 1st day of rewatering (1D RH), and on the 7th day of rewatering (7D RH). Error bars represent the standard errors. Homogeneous groups are denoted by the same letter according to the Fisher-LSD test (*p* = 0.05). HDT, high-drought-tolerant form; LDT, low-drought-tolerant introgression form; C, control introgression forms not treated with PTIO. PTIO, introgression forms treated with PTIO.

Statistically significant differences in gas exchange parameters between the plants in the control conditions and the plants cultivated under water deficit conditions started to be visible on the 12th day of the experiment. Dynamic changes of net photosynthesis rate (P_N_) were observed in both introgression forms, depending on both drought and PTIO treatment. In PTIO non-treated forms, it decreased after 12 days of water deficit, whereas it increased in the HDT treated with PTIO (~20%). However, after 14 days of water deficit, the situation was opposite and the PTIO-treated plants were characterized by a lower photosynthesis rate than the non-treated ones, but the difference was significant only for the PTIO-LDT form (~−37%). After 7 days of rewatering, P_N_ increased significantly in all the experimental groups of plants (**Figure 4A**, **Supplementary Table S2**). Stomatal conductance (*g_s_*) (**Figure 4B**) and transpiration (E) (**Figure 4C**) were higher in both groups of LDT plants under control conditions. During water deficit, these parameters decreased, especially in the PTIO non-treated plants just after 12 days, and the differences were statistically significant, compared to the PTIO-treated plants. After 14 days, *g_s_* and E values were the lowest, but no differences with statistical significance could be observed among the experimental groups. During rewatering, *g_s_* recovered faster in the LDT form; however, after 7 days of rewatering, the control condition values of *g_s_* and E were achieved only by the form HDT not treated with PTIO. Significant differences in C*_i_*, compared to control values, were observed after 1 day of rewatering in the PTIO-treated plants: the HDT form was characterized by a decrease of this parameter, and the LDT form was characterized by an increase (**Figure 4D**, **Supplementary Table S2**).

### 3.6 Chlorophyll fluorescence

The F_v_/F_m_ parameter decreased under drought and after 1 day of rewatering in all the analyzed plant groups. This parameter recovered slightly after 7 days of rewatering but reached the control values only in the LDT genotype (**Figure 4E**, **Supplementary Table S2**).

### 3.7 Activity of chloroplastic fructose-1,6-bisphosphate aldolase

In the control conditions, the activity of pFBA was higher in both the LDT groups. After 14 days of water deficit, it decreased in both forms, but significant differences were observed only between the HDT-PTIO and LDT-PTIO plants, and the LDT form was characterized by a higher aldolase activity than the HDT (~90%). After 7 days of rewatering, only the PTIO-treated introgression forms recovered aldolase activity to the level observed in the control conditions (**Figure 4F**, **Supplementary Table S2**).

### 3.8 Reactive oxygen species content

**Figure 5:**
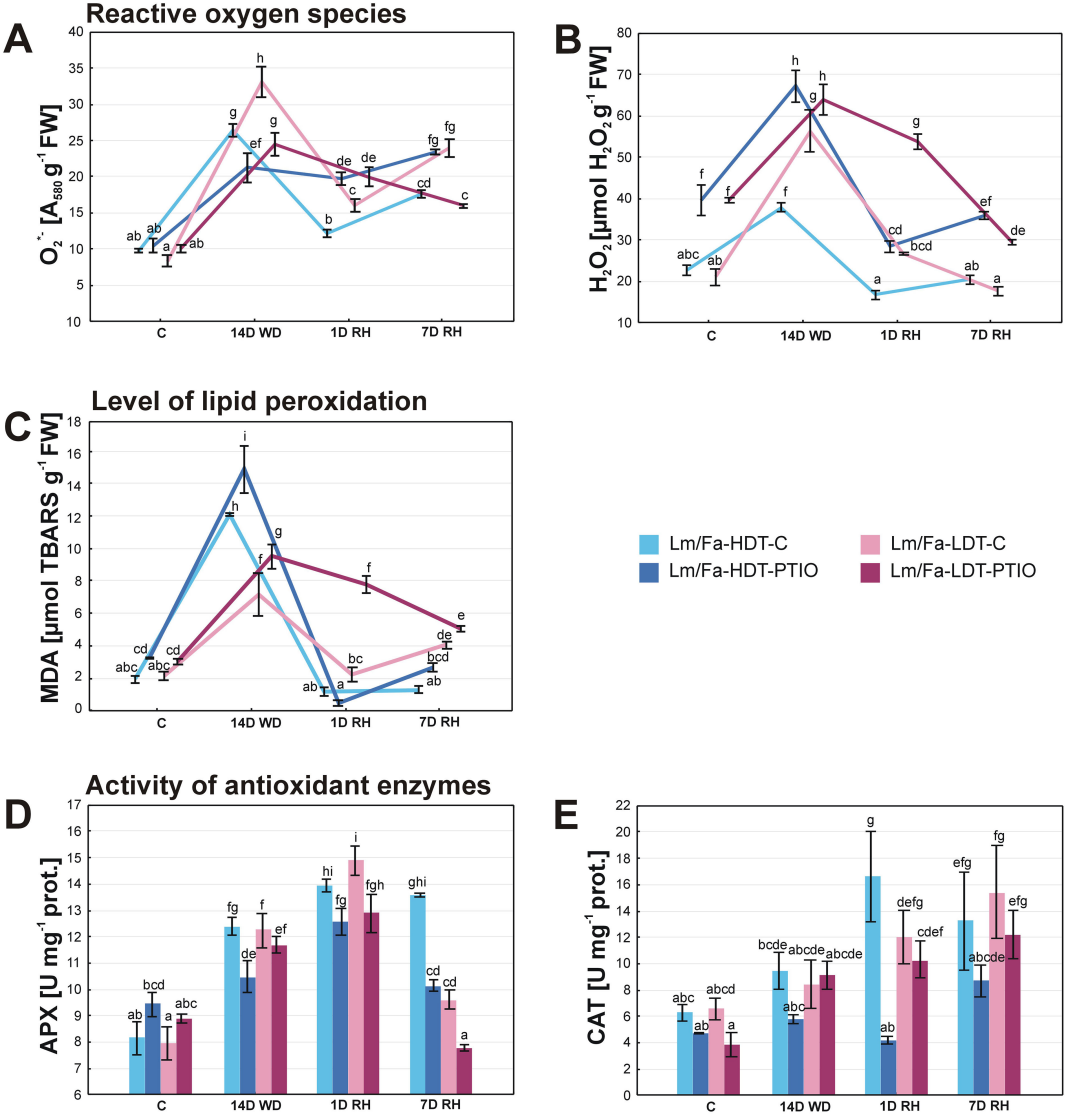
Reactive oxygen species content: superoxide anion radical (O_2_^•−^) **(A)** and hydrogen peroxide (H_2_O_2_) (**B**), malondialdehyde (MDA) content **(C)**, and activity of antioxidant enzymes: ascorbate peroxidase (APX) **(D)** and catalase (CAT) **(E)** in the analyzed introgression forms in control conditions (C), on the 14th day of water deficit (14D WD), on the 1st day of rewatering (1D RH), and on the 7th day of rewatering (7D RH). Error bars represent the standard errors. Homogeneous groups are denoted by the same letter according to the Fisher-LSD test (*p* = 0.05). HDT, high-drought-tolerant form; LDT, low-drought-tolerant introgression form; C, control introgression forms not treated with PTIO; PTIO, introgression forms treated with PTIO.

With respect to O_2_^•−^ content, clear differences between the control plants were observed on the 14th day of water deficit and after rewatering with a higher content of this molecule in the LDT form. Drought significantly increased O_2_^•−^ content in both forms; however, after the initiation of rewatering, it was reduced, whereas it increased slightly after 7 days of rewatering. PTIO treatment significantly reduced O_2_^•−^ content in both forms under drought, but after the initiation of rewatering, it had the opposite effect. After 7 days of rewatering, the PTIO-treated HDT form and the non-treated plants increased slightly the content of O_2_^•−^. Meanwhile, the PTIO-treated LDT form decreased the level of this parameter (**Figure 5A**, **Supplementary Table S2**).

Water deficit conditions significantly increased H_2_O_2_ content in both Lm/Fa forms, but it was higher in the LDT (~168%). After rewatering, H_2_O_2_ content decreased to the values observed in control conditions for both control genotypes. PTIO treatment significantly elevated H_2_O_2_ content in both forms in all the analyzed time points (**Figure 5B**, **Supplementary Table S2**).

### 3.9 Level of lipid peroxidation

The malondialdehyde content was determined based on the level of TBARS. The TBARS level increased under drought in both control groups of plants, but more significantly in the HDT form (~522%). Full recovery of this parameter was observed after the initiation of rewatering in both forms, but after 7 days of rewatering, it increased again in the LDT form (~91%). PTIO treatment of the plants significantly elevated TBARS content in both forms under drought and in the LDT form at the beginning of the recovery period (**Figure 5C**, **Supplementary Table S2**).

### 3.10 Activity of the antioxidant enzymes

Drought induced the activity of APX in a similar way for both control plants. Moreover, the activity of this enzyme increased even higher after the initiation of recovery. After 7 days of rewatering, it decreased but only in the LDT form. PTIO treatment significantly reduced APX activity in the HDT form on the 14th day of water deficit (~−15%) and 7 days of recovery (~–25%) and in the LDT form but only under recovery (~−19%) (**Figure 5D**, **Supplementary Table S2**).

Catalase activity increased significantly during the whole period of rewatering in the HDT form and after 7 days of rewatering in the LDT form (~135%), compared to the control values. PTIO treatment reduced CAT activity during the experiment, but this phenomenon was statistically significant only after the initiation of rewatering in the HDT form (~−75%), compared to the control plants (**Figure 5E**, **Supplementary Table S2**).

## 4 Discussion

Various strategies are used to regulate NO content in plant tissues in order to recognize its role in plant response to environmental conditions. Generally, exogenous NO was applied to plants in the form of sodium nitroprusside (SNP) or nitrosoglutathione (GSNO) leading to tissue NO saturation and sometimes overaccumulation (García-Mata and Lamattina, 2001; Neill et al., 2002; Guo et al., 2003; Casaretto et al., 2021; Lozano-Juste and León, 2010). These conditions could result in homeostasis imbalance and lead to nitrosative stress. On the other hand, exogenous NO has been demonstrated to exhibit remarkable drought tolerance-enhancing properties, significantly modulating stomatal movement and reducing oxidative stress. Supplementation with NO in the form of different donor compounds can also significantly affect root structure, photosynthesis, osmolyte accumulation, and plant seed formation under drought conditions (Saini et al., 2024). Less common was a modulation of NO content by using NO scavengers such as cPTIO or PTIO (García-Mata and Lamattina, 2002; Wu et al., 2017). PTIO was reported previously to be an efficient scavenger of NO produced during water deficit for *Triticum aestivum* (Wu et al., 2017) and also for *Festuca* species (Perlikowski et al., 2022). Moreover, the PTIO application does not affect the enzymatic sources of NO in cells, such as the NOS protein (Maeda et al., 1994). The results obtained in our experiments clearly indicated that the application of PTIO in the form of watering and spraying of plants significantly reduced the generation of NO under the analyzed experimental conditions. However, compared to the control values, it can be concluded that this reduction was not complete and NO was still generated at low levels during the experiment. Moreover, it is worth mentioning that the LDT introgression form accumulated significantly higher NO levels under stress conditions (**Figure 2B**), which was also observed in our earlier study on *F. arundinacea* and *F. glaucescens* (Perlikowski et al., 2022). This phenomenon might be directly associated with a higher sensitivity of LDT plants to water deficit, and overaccumulation of NO might be involved in the strategy to cope with such stress conditions.

### 4.1 Nitric oxide is required in the early response of plants to water deficit and stomata closure

The initial reaction of plants exposed to water deficit is a decrease in transpiration rate due to ABA-triggered stomatal closure. This mechanism effectively diminishes stomatal conductance to maintain high water potential in the leaves, thereby preventing dehydration (Tardieu, 2005). Nonetheless, the reduction in stomatal conductance also restricts the diffusion of CO_2_ into the mesophyll, resulting in CO_2_ assimilation rate and overall efficiency of photosynthesis decline (Flexas et al., 2004). A lower efficiency of photosynthesis was observed in the analyzed plants on the 14th day of water deficit with respect to P_N_, F_v_/F_m_, and the activity of chloroplast aldolase (**Figures 4A,E,F**). Here, also on the 14th day of water deficit conditions, both Lm/Fa introgression forms reduced their RWC and increased EL, but it was observed to have a higher degree in the LDT form (**Figures 3A,B**). This phenomenon had also been noticed in our earlier experiments (Perlikowski et al., 2014). However, PTIO treatment and the consequent NO deficiency did not have any direct significant impacts on the RWC and EL parameters in Lm/Fa, as it was previously reported for other forage grasses, such as *F. arundinacea* and *F. glaucescens* (Perlikowski et al., 2022). Only the EL parameter decreased slightly more after rewatering in the LDT form treated with PTIO, compared to the genotype without NO deficiency. This is in contradiction to our previous findings, indicating that PTIO treatment had negative effects on membrane stability in *Festuca* plants after rewatering (Perlikowski et al., 2022). Earlier findings suggested that an elevated NO content could help preserve water status in *Nicotiana tabacum* and *T. aestivum* during osmotic stress (Tan et al., 2008). It also enabled a reduced consumption of water in *T. aestivum* during polyethylene glycol (PEG) treatment. This effect was reversed following cPTIO application (Xing et al., 2004). The findings support our observations, indicating that both the PTIO-treated Lm/Fa forms had significantly higher water usage during early stages of soil water deficit progression. The increase in water uptake was more visible in the LDT form. This phenomenon, which has a high probability, made it more susceptible to dehydration effects.

Earlier research on NO functions under drought conditions suggested that this gaseous product of plant metabolism is necessary to trigger the ABA-dependent transduction pathway of drought response for a positive regulation of ABA (García-Mata and Lamattina, 2001; Guo et al., 2003; Desikan et al., 2002). However, the other authors contradicted this theory, suggesting that NO is not necessary for stomatal closure in dependence on ABA (Lozano-Juste and León, 2010; León et al., 2014). Our results indicate that NO scavenging could lead to increased accumulation of ABA, but this effect was visible only in the LDT form on the 14th day of water deficit. This observation at least partially corresponds to the results achieved for *F. arundinacea* and *F. glaucescens* (Perlikowski et al., 2022). For the HDT form, a similar accumulation of ABA in PTIO-treated and non-treated plants was observed. On the other hand, stomatal aperture was less responsive on the 12th day of water deficit conditions under PTIO treatment, but on the 14th day of water deficit, stomatal conductance was significantly reduced in both groups of plants (**Figures 4A–C**). The results obtained for gas exchange parameters, including the assimilation of CO_2_, corresponded well with our previous observations (Perlikowski et al., 2022), at least in less severe water deficit conditions up to the 12th day of water deficit, indicating that NO deficiency significantly prevented a proper response of stomatal aperture to water deficit resulting in higher transpiration and assimilation of CO_2_ in both Lm/Fa introgression forms. The situation changed after 14 days of water deficit, when NO-deficient plants decreased *g_s_*, *E*, and P_N_ to the levels comparable to those observed in the forms not treated with PTIO. However, such a situation was not observed in *Festuca* species before (Perlikowski et al., 2022). This indicates that in more severe water deficit conditions, NO might not be required for stomatal closure, and the differences between the analyzed introgression forms and the analyzed earlier *Festuca* species corresponded to different drought tolerance capacity of these two groups of plants. It might not be excluded that a similar response to more severe water deficit conditions would be observed for these two groups if the drought period was prolonged above 14 days in the case of *Festuca* species. Plants treated with PTIO were characterized by increased CO_2_ assimilation rate in the control and on the 12th day of water deficit which was associated with higher stomatal conductance (**Figures 4A,B**). This observation was supported by another study that revealed how the external implementation of NO in the form of SNP treatment triggered stomatal closure (García-Mata and Lamattina, 2001). In the research on *Vicia faba* and *Pisum sativum*, NO was recognized as a crucial component accountable for ABA-triggered stomatal closure (García-Mata and Lamattina, 2002), highlighting ABA’s role in stimulating NO synthesis. Furthermore, the use of NO scavenger, such as cPTIO, notably diminished stomatal closure in the presence of ABA (García-Mata and Lamattina, 2003). This suggests that ABA alone may not be sufficient for effective signaling stress conditions, particularly during the initial phases of drought when a relatively high content of NO may be required to prompt rapid stomatal closure. On the other hand, some researchers have suggested that NO acts as a suppressor of ABA function (Casaretto et al., 2021), with its presence promoting stomatal opening during the initial phases of drought in *Glycine max*. However, the deficiency of NO during the later stages of drought may not be sufficient to prevent stomatal closure (Wang et al., 2015). Furthermore, some observations have shown that the application of very high amounts of NO resulted in stomatal opening even under exogenous ABA treatment (Sakihama et al., 2003). Therefore, it is proposed that an elevated concentration of NO is responsible for stomatal closure, while surpassing this threshold could have the opposite effect (Sokolovski and Blatt, 2004). Moreover, there is a suggestion that NO may function independently of ABA and regulates stomatal aperture through a distinct pathway (van Meeteren et al., 2020).

Previously, it was documented that high concentrations of NO notably reduced the photosynthesis rate in *Avena sativa* and *Medicago sativa* plants (Hill and Bennett, 1970). Nevertheless, the impact of exogenous NO on this process remains uncertain, as plant treatment with SNP had a concomitant negative effect on photosynthetic enzymes in *T. aestivum* or *Phaseolus aureus* (Leshem, 2001; Lum et al., 2005), but also enhanced photosynthesis rate in *Lycopersicon esculentum* or cucumber (Fan et al., 2007; Hayat et al., 2011). The reduction in photosynthesis was primarily attributed to stomatal closure triggered by the activity of NO (Neill et al., 2002). Nevertheless, a notable impact of exogenous SNP application on photosynthetic enzymes, such as the inhibition of Rubisco and Rubisco activase through S-nitrosylation, has also to be acknowledged (Lum et al., 2005; Abat et al., 2008). Furthermore, the process of tyrosine nitration or S-glutathionylation was identified as a mechanism for regulating the activity of 1,6-bisphosphate aldolase and other photosynthetic enzymes during salinity stress (Tanou et al., 2012; Zaffagnini et al., 2012). These results are consistent with our discovery of increased aldolase activity in plants treated with PTIO during the recovery phase (**Figures 4F**), suggesting that a reduced NO content may indeed enhance photosynthesis by influencing the Calvin cycle. This phenomenon requires further studies.

### 4.2 Nitric oxide supports hydrogen peroxide scavenging

The generation of ROS in plant cells represents a key consequence of plants being exposed to unfavorable environmental circumstances, such as periods of water deficit (Lee et al., 2012). ROS can play a dual role in plant cells. On one hand, ROS may act as signaling molecules that enhance the plant’s response to stress. On the other hand, it has the potential to disrupt the cellular redox balance, leading to oxidative stress that results in cellular damage, including irreversible changes to enzymes, lipid peroxidation, and DNA damage (Richards et al., 2015; Huang et al., 2019). Differentiating between these two functions of ROS is challenging, as they often occur simultaneously within the cellular environment (Huang et al., 2019). It is widely recognized that unfavorable environmental conditions trigger an excess production of O_2_^•−^ and H_2_O_2_, leading to increased lipid peroxidation, as indicated by elevated levels of MDA (Ali et al., 2018; Habib et al., 2020).

It was indicated earlier that elevated content of NO from exogenous sources such as SNP leads to increased MDA detoxification under salt stress in *F. arundinacea* (Li et al., 2016) and also *T. aestivum* under drought conditions (Habib et al., 2020). This finding corresponds well with our results obtained for both introgression forms with deprived NO, which had elevated MDA levels on the 14th day of water deficit and after the beginning of rewatering in the LDT form. Similar results were observed in our earlier research on *F. arundinacea* and *F. glaucescens* (Perlikowski et al., 2022). However, in the present research, this effect was not associated with membrane damage evaluated by the EL parameter.

The production of O_2_^•−^ was induced under drought in the analyzed Lm/Fa forms, but what is surprising, especially in plants, characterized by higher NO generation (**Figure 5A**). This finding is contrary to our previous research, where higher O_2_^•−^ generation was promoted in *F. arundinacea* and *F. glaucescens*, characterized by NO depletion (Perlikowski et al., 2022). Moreover, other authors also suggested that NO deficiency induced O_2_^•−^ accumulation under salt stress in *F. arundinacea* (Li et al., 2016). As we suggested earlier (Perlikowski et al., 2022), enhanced generation of O_2_^•−^ could be associated with insufficient ONOO¯ formation and lower protein nitration in *Festuca* genotypes; herein, we observed a decreased O_2_^•−^ generation. The balanced level of ONOO¯-mediated tyrosine nitration in proteins might be associated with many regulatory processes (Gzyl et al., 2016). However, an initial stage of rewatering had an opposite effect than drought, indicating that a deficiency of NO in plants had no effect on O_2_^•−^ content, while a saturation with NO in plants significantly decreased O_2_^•−^ content (**Figure 5A**). This phenomenon partially corresponds with our earlier observations (Perlikowski et al., 2022).

Our previous research did not provide the univocal results for H_2_O_2_ accumulation in different NO contents under drought, since only the HDT plants were characterized by higher H_2_O_2_ generation under NO scarcity (Perlikowski et al., 2022). Herein, both Lm/Fa introgression forms accumulated higher amounts of H_2_O_2_ on the 14th day of water deficit, compared to the control, but the differences between PTIO-treated and non-treated plants were higher in the HDT form. Similar results were also indicated by other authors for *F. arundinacea*, but with respect to salt stress (Li et al., 2016). Moreover, after the initiation of rewatering, the elevated level of H_2_O_2_ was still observed in NO-depleted plants, especially in the LDT (**Figure 5B**). We suggest that this phenomenon might be associated at least partially with impaired activity of antioxidant enzymes observed under NO deficiency in the analyzed plants, since this correlation was also well visible in previous experiments (Perlikowski et al., 2022). It was suggested that the cellular production of NO and H_2_O_2_ could boost ABA-mediated drought tolerance and enhance the activity of antioxidant enzymes (Luna et al., 2005). A reduction in H_2_O_2_ content following SNP treatment had been previously documented in *Discorea opposita* (Seregelyes et al., 2003). Nitric oxide could potentially activate the antioxidant system to modulate H_2_O_2_ content (Zeng et al., 2011). Herein, a negative effect of NO scavenging on APX activity could be noticed under drought and recovery in the analyzed introgression forms. The obtained results, however, did not fully correspond to our previous findings, which indicated that NO deficiency had a stimulating effect on the antioxidant system, especially in *F. glaucescens* (Perlikowski et al., 2022). Previous research demonstrated that the application of SNPs can notably enhance the activity of the antioxidant system under drought, encompassing CAT, superoxide dismutase (SOD), and peroxidases under drought treatment (Moussa and Mohamed, 2011). Increasing evidence suggests that NO has the potential to regulate the activity of antioxidant enzymes through the modification of specific amino acids. Nitration of tyrosine was able to inhibit APX activity in cotton (*Gossypium hisutum*) (Kong et al., 2016). On the other hand, S-nitrosylation of APX could enhance the plant tolerance to oxidative stress (Astier et al., 2011).

## 5 Conclusions

The comparison of LDT and HDT introgression forms indicated a significant distinction in the production of NO under stress conditions. A less tolerant form accumulated an elevated amount of NO, possibly due to its enhanced susceptibility to stress conditions.

The PTIO-treated plants in the case of both Lm/Fa introgression forms had significantly higher water uptake during the early stages of water deficit progression. The increase in water uptake was more visible in the LDT form. With a high probability, this phenomenon made it more vulnerable to dehydration effects.

An improvement of photosynthetic capacity manifested by higher CO_2_ assimilation levels in plants treated with PTIO on the 12th day of water deficit was observed. This phenomenon was associated with delayed stomata closure observed under NO deficiency in these plants.

Delayed stomata closure observed on the 12th day of water deficit in PTIO-treated plants resulted also in increased transpiration at this time point, which in fact could negatively affect these plants.

The treatment of plants with PTIO resulted in several adverse consequences in the analyzed Lm/Fa introgression forms, including elevated H_2_O_2_ accumulation, decreased activity of APX on the 14th day of water deficit and recovery, and higher lipid peroxidation, which could impact cellular homeostasis of the analyzed plants.

## Data Availability

The original contributions presented in the study are included in the article/**Supplementary Material**. Further inquiries can be directed to the corresponding author.
